# In Vitro Antibiofilm Activity of Fosfomycin Alone and in Combination with Other Antibiotics against Multidrug-Resistant and Extensively Drug-Resistant *Pseudomonas aeruginosa*

**DOI:** 10.3390/ph17060769

**Published:** 2024-06-12

**Authors:** Mia Slade-Vitković, Ivanka Batarilo, Luka Bielen, Gordana Maravić-Vlahoviček, Branka Bedenić

**Affiliations:** 1 Microbiology Department, Croatian Institute of Transfusion Medicine, 10000 Zagreb, Croatia; mia.slade@gmail.com (M.S.-V.); ivanka.batarilo@hztm.hr (I.B.); 2 Department of Internal Medicine, Clinical Hospital Centre Zagreb, 10000 Zagreb, Croatia; luka.bielen@yahoo.com; 3 Department of Internal Medicine, School of Medicine, University of Zagreb, 10000 Zagreb, Croatia; 4 Department of Biochemistry and Molecular Biology, Faculty of Pharmacy and Biochemistry, University of Zagreb, 10000 Zagreb, Croatia; 5 Clinical Department for Clinical and Molecular Microbiology, Clinical Hospital Centre Zagreb, 10000 Zagreb, Croatia; 6 Biomedical Research Center Šalata—BIMIS, School of Medicine, University of Zagreb, 10000 Zagreb, Croatia

**Keywords:** *Pseudomonas aeruginosa*, multidrug-resistant, extensively drug-resistant, fosfomycin, antibiotic combinations, biofilm, inhibition of biofilm formation, biofilm disruption

## Abstract

Background: Due to its rapid resistance development and ability to form biofilms, treatment of *Pseudomonas aeruginosa* infections is becoming more complicated by the day. Drug combinations may help reduce both resistance and biofilm formation. Methods: Using the microtiter plate assay, we investigated the in vitro inhibition of biofilm formation and the disruption of preformed biofilms in multidrug-resistant and extensively drug-resistant clinical isolates of *P. aeruginosa* in the presence of peak plasma levels of eight antipseudomonal antibiotics alone and in combination with fosfomycin: ceftazidime, piperacillin/tazobactam, cefepime, imipenem, gentamicin, amikacin, ciprofloxacin and colistin. Results: Combination therapy was significantly superior to monotherapy in its inhibition of biofilm formation. The highest inhibition rates were observed for combinations with colistin, cefepime and ceftazidime. Conclusion: Our results support fosfomycin combination therapy as an enhanced prophylactic option. Moreover, combinations with β-lactam antibiotics and colistin demonstrated a more potent inhibition effect on biofilm formation than protein synthesis inhibitors.

## 1. Introduction

*Pseudomonas aeruginosa* causes various infections, especially in individuals with impaired immune systems or underlying health conditions, such as respiratory tract infections; urinary tract infections; skin and soft tissue infections; wound, ear and eye infections; sepsis or bacteraemia. It is considered one of the most important hospital-acquired pathogens, and its treatment is becoming more difficult by the day as antibiotic resistance increases. Severe *P. aeruginosa* infections are link to high mortality rates and therefore require empirical treatment combinations with different mechanisms of action [1]. *P. aeruginosa* is also known for its ability to form biofilms on human tissue, as well as on various surfaces, such as catheters and different prosthetic devices. Biofilms provide a safe harbour for bacteria, where they can withstand harsh conditions, escape the host’s immune system and antibiotics and be the source of persistent and recurrent infections. Bacteria in biofilms exhibit different behaviour than their planktonic forms. Antibiotics inhibit bacterial growth or kill planktonic bacteria but are much less effective against sessile biofilm bacterial cells, so-called persisters [2]. One of the most important virulence factors of *P. aeruginosa* is biofilm formation [3]. Biofilm elimination is very difficult and requires high doses of antibiotics, often in combination, with very uncertain outcomes [4,5]. Nearly 80% of persistent bacterial infections are estimated to be linked to biofilms [6], and biofilm-related *P. aeruginosa* infections often lead to treatment failure [2].

Although antibiotics are more useful in preventing biofilm formation, some data also suggest a reduction in the biofilm matrix in the presence of antibiotics, proposing different mechanisms [7,8,9]. Aminoglycosides showed a preventive effect on the early adhesion of *P. aeruginosa* isolates. Several antibiotics, including ceftazidime, piperacillin/tazobactam and colistin, have shown some effect against bacterial adhesion, motility and biofilm formation [2]. Fosfomycin has recently reemerged as an interesting treatment option for antimicrobial-resistant infections, as no cross-resistance and low resistance rates are expected [10,11,12]. It has been shown to penetrate very well into mature biofilms formed by *P. aeruginosa* [13,14] and to enhance the penetration of other antibiotics [15,16], presumably due to its low molecular weight [13,14,17,18]. Fosfomycin has also been associated with biofilm reduction in uropathogenic *E. coli* [18].

Fosfomycin monotherapy is of concern because of rapid mutant selection; therefore, dual antimicrobial coverage is often advised [12,19,20]. Moreover, since monotherapy is usually unsuccessful in eradicating biofilms, combination therapy is an important treatment alternative, and it is necessary to investigate the possible anti-biofilm activities of antibiotics, alone or in combination [3,4,5,16,21]. Chronic *P. aeruginosa* infections in cystic fibrosis are very difficult to treat, but aggressive antibiotic therapy approaches have recently been proven successful in eradicating new *P. aeruginosa* colonisations [22]. Several antimicrobial combinations have been shown to be synergistic against *P. aeruginosa* biofilms [4,5]. However, studies on combination therapy with fosfomycin on *P. aeruginosa* biofilms are limited [21,23,24,25,26,27,28] and have mostly been carried out on a few isolates, using a small number of antibiotic combinations [16,25,27,28,29]. Fosfomycin synergy against *P. aeruginosa* biofilms has mainly been recorded in combination with aminoglycosides and quinolones [21,23,24,25,26,27,28]. Biofilm eradication has also been reported in combination with tobramycin [16]. Little is documented on the effects of combining fosfomycin with colistin and β-lactam antibiotics. Synergy has been observed in combination with high doses of imipenem, but this is of questionable clinical value due to the potential toxicity of the drug levels required for clinical effect [3]. Recently, synergy has also been documented in combination with colistin against *P. aeruginosa* biofilms at concentrations achievable through the inhalation of nebulised drugs [30]. However, to our knowledge, no reports covering combinations with the β-lactam antibiotics ceftazidime, cefepime or piperacillin/tazobactam against extensively drug-resistant (XDR) isolates have been published.

Therefore, the aim of our research was to analyse the in vitro inhibition of biofilm formation, as well as the disruption of preformed biofilms, in clinical isolates of *P. aeruginosa* with various resistance traits, including multidrug-resistant (MDR) and extensively drug-resistant (XDR) isolates, in the presence of peak plasma levels of eight routinely used antipseudomonal antibiotics alone or in combination with fosfomycin: β-lactam antibiotics, which inhibit peptidoglycan synthesis (ceftazidime, cefepime, piperacillin/tazobactam, imipenem); fluoroquinolones, which block DNA synthesis (ciprofloxacin); aminoglycosides, which prevent protein synthesis (gentamicin, amikacin) and polypeptides (colistin), interfering with outer membrane and cytoplasmic membrane function.

## 2. Results

### 2.1. Antibiotic Susceptibility

For this study, 42% multidrug-resistant isolates (*n* = 18) and 58% extensively drug-resistant isolates (*n* = 25) were used. Resistance to fosfomycin was determined in 51% of the isolates (*n* = 22). The resistance rates to the other antibiotics were as follows: colistin 14% (*n* = 6), piperacillin/tazobactam 40% (*n* = 17), amikacin 47% (*n* = 20), cefepime 72% (*n* = 31), gentamicin 79% (*n* = 34), ceftazidime 86% (*n* = 37), ciprofloxacin 88% (*n* = 38), meropenem 91% (*n* = 39), imipenem 91% (*n* = 39). The MIC values of the antibiotics tested are listed in Table 1.

### 2.2. Detection of Biofilm Formation

All tested clinical isolates were biofilm producers according to the microtiter plate assay. Of the 43 isolates, 32.6% (*n* = 14) were strong biofilm producers (SBPs), 41.9% (*n* = 18) were moderate biofilm producers (MBPs) and 25.6% (*n* = 11) were weak biofilm producers (WBPs).

### 2.3. Inhibition of Biofilm Formation

For the inhibition of biofilm formation (IBF) test, 24 isolates were used, of which 13 were strong and 11 were moderate biofilm producers. Each isolate was exposed to peak plasma concentrations of the antibiotics alone and in combination.

**Figure 1:**
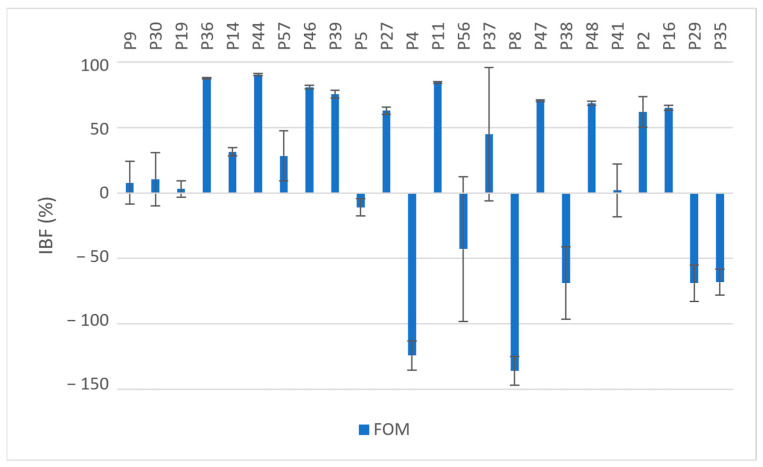
Inhibition of biofilm formation (IBF, %) in MDR and XDR isolates (P9–P35) by fosfomycin (Fom); values expressed as means with SD.

Exposure to fosfomycin resulted in varying degrees of inhibition in 70.8% (*n* = 17) of the isolates and ranged from 2.09% to 90.43%. A higher OD_620_ value after exposure to fosfomycin was observed in 29.2% of the isolates (*n* = 7) compared to the OD_620_ values of the unexposed isolates; see Figure 1. These seven isolates were all resistant to fosfomycin (MIC ≥ 64 µg/mL). Five of these seven isolates were MBPs when not exposed to fosfomycin but were converted into SBPs upon exposure. The overall mean IBF value obtained was 14.88%; see Table 2. There was no statistically significant difference between the OD_620_ values with and without exposure to fosfomycin (*p* = 0.069).

**Figure 2:**
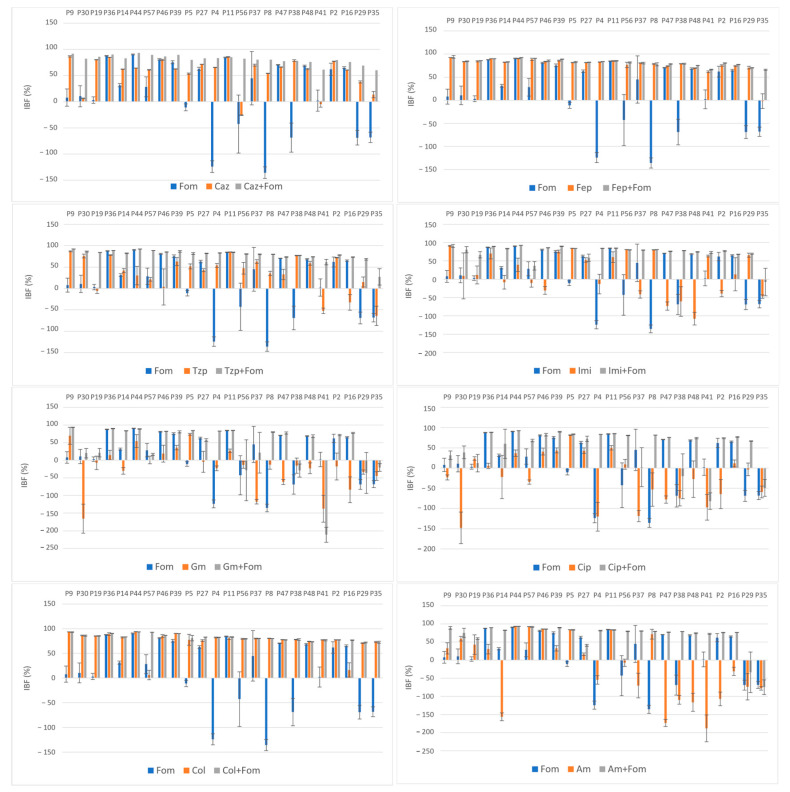
Effect of single and combined antibiotic treatments on biofilm development of MDR and XDR isolates (P9–P35); inhibition of biofilm formation (IBF) percentage values expressed as means with standard deviations (SDs) obtained with the crystal violet assay. Abbreviations: fosfomycin (FOM), ceftazidime (CAZ), piperacillin/tazobactam (TZP), cefepime (FEP), imipenem (IMI), gentamicin (GM), amikacin (AM), ciprofloxacin (CIP) and colistin (COL).

The average obtained IBF values of the antibiotics alone and in combination are listed in Table 2. The effect of single and combined antibiotic treatments on IBF is shown graphically in Figure 2. While large differences were observed among the inhibition properties of individual antibiotics, a more uniform inhibition pattern was observed when these antibiotics were used in combination with fosfomycin. The highest inhibition rates were observed with colistin alone and in combination, a result that was somewhat expected because of its low resistance rates (14%), and with cefepime alone and in combination, despite high resistance rates to both cefepime (72%) and fosfomycin (51%).

**Figure 3:**
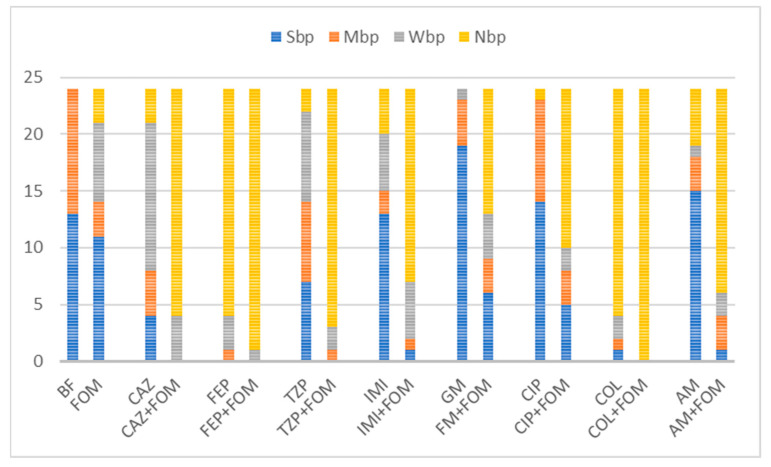
Distribution of biofilm production categories among 24 isolates unexposed and exposed to single or combined antibiotics. Abbreviations: fosfomycin (FOM), ceftazidime (CAZ), piperacillin/tazobactam (TZP), cefepime (FEP), imipenem (IMI), gentamicin (GM), amikacin (AM), ciprofloxacin (CIP), colistin (COL), non-biofilm producer (NBP), weak biofilm producer (WBP), moderate biofilm producer (MBP), strong biofilm producer (SBP) and biofilm formation of unexposed isolates (BF). Combinations with fosfomycin yielded downgrades in category with a larger fraction of non-biofilm producers among tested isolates. Out of 13 strong and 11 moderate biofilm producers, exposure to antibiotic combinations with fosfomycin resulted in inhibition of biofilm formation in all combinations with colistin (*n* = 24, 100%), 95.8% (*n* = 23) with cefepime, 87.5% (*n* = 21) with piperacillin/tazobactam, 83.3% (*n* = 20) with ceftazidime, 75% (*n* = 18) with amikacin, 70.8% (*n* = 17) with imipenem, 58.3% with ciprofloxacin (*n* = 14) and 45.8% (*n* = 11) with gentamicin.

Although the highest values of IBF were recorded with combinations with colistin, with a mean inhibition percentage of 82.3%, no statistical significance was found compared to the use of colistin alone (*p* = 0.47). The use of colistin alone (75.6%) and in combination (82.3%) was superior to the use of fosfomycin (14.9%) alone in terms of the IBF. Nevertheless, comparison of the biofilm production categories showed the superiority of combination therapy, with all the isolates belonging to the NBP category because of the colistin–fosfomycin combination; see Figure 3. High IBF levels were also observed with cefepime alone and in combination. However, combination therapy was superior to the use of single antibiotics in terms of category change, as combination therapy resulted in a higher percentage of NBPs; see Figure 3. All the other antibiotic combinations, with ceftazidime, piperacillin/tazobactam, imipenem, gentamicin, ciprofloxacin and amikacin, resulted in significant IBF levels compared to single use (*p* < 0.05). In addition, all antibiotic combinations led to a higher proportion of NBPs; see Figure 3.

High average IBF values were also found for combinations with the β-lactam antibiotics ceftazidime (81%), piperacillin/tazobactam (78.8%) and imipenem (74.1%). The IBF of combinations with ceftazidime, piperacillin/tazobactam and imipenem was significantly higher compared to that with single use of antibiotics (56.5%, 36.8% and 15.2%, respectively), Table 2. Moreover, all the antibiotic combinations resulted in higher proportions of NBPs; see Figure 3. The use of imipenem alone resulted in the IBF in 58.3% of the isolates (*n* = 14), with a mean of 15.2%. The measured OD_620_ values of 10 isolates were higher when imipenem alone was used compared to the measured OD_620_ biofilm values of the unexposed controls, resulting in negative IBF values. Of these isolates, 70% (*n* = 7) had a MIC_IMI_ value of ≥64 and were thus exposed to subMIC concentrations of imipenem. A similar effect was observed when gentamicin, amikacin and ciprofloxacin were used alone. A total of 93.8% of isolates (15/16) with negative IBF values were exposed to subMIC concentrations of gentamicin, 100% of isolates (14/14) with negative IBF values were exposed to subMIC concentrations of colistin and 66.7% isolates (8/12) with negative IBF values were exposed to subMIC concentrations of amikacin. While the mean values of the IBF of gentamicin (−20.8%), amikacin (−18.3%) and ciprofloxacin (−24.2%) were low, combination therapy with fosfomycin resulted in significantly higher IBF values (39.9%, 68.1% and 52.8%, respectively). Additionally, these antibiotic combinations also led to a higher proportion of NBPs as well; see Figure 3.

### 2.4. Disruption of Preformed Biofilms

The disruption of biofilms was investigated in 24 h old biofilms from eight strong biofilm producers. The preformed biofilms were exposed to antibiotics for 6 h and 24 h. Neither a statistically significant reduction in biofilms nor a change in OD_620_ value was observed, regardless of the duration of antibiotic exposure; see Table 3 and Table 4. Nevertheless, a category change from strong to moderate biofilm producers was observed in some isolates after 6 h of antibiotic exposure. This effect was observed when the P14 biofilms were exposed to fosfomycin alone, ceftazidime and ciprofloxacin in combination and cefepime, imipenem, gentamicin, colistin and amikacin alone and in combination. The same effect was also noted after the P46 biofilms were exposed to imipenem alone and in combination and after the P36 biofilms were exposed to amikacin in combination. Similarly, 24 h of antibiotic exposure resulted in equal category downgrading, from strong to moderate, when the P36 biofilms were exposed to amikacin, colistin and ciprofloxacin in combination and piperacillin/tazobactam alone.

## 3. Discussion

Our results showed neither an inhibitory nor a destructive effect of fosfomycin alone on MDR and XDR *P. aeruginosa* biofilms. Wang et al. showed a similar result, with no anti-biofilm effects on fosfomycin-susceptible *P. aeruginosa* isolates, despite the high antibiotic concentrations used (up to 1.024 μg/mL) [21]. Fosfomycin inhibits cell wall synthesis and works best against rapidly growing bacteria, which could partly explain these results.

Weak β-lactam antibiofilm activity was anticipated due to the slow growth of biofilm bacteria, which hinders β-lactam activity targeting the peptidoglycan synthesis of actively growing and dividing bacteria [31]. Nevertheless, high IBF values were achieved in our study in combination with β-lactam antibiotics. This is the first report, to the best of our knowledge, of fosfomycin combinations with the β-lactam antibiotics ceftazidime, cefepime and piperacillin/tazobactam against *P. aeruginosa* biofilm. Here, we have shown, for the first time, that β-lactam antibiotics have a more potent IBF effect in comparison to protein synthesis inhibitors and quinolones, even when not combined with fosfomycin. Moreover, we observed that the IBF effect was most pronounced in combinations with β-lactam antibiotics (ceftazidime, cefepime) and polypeptides (colistin) despite high resistance rates to ceftazidime, cefepime and fosfomycin. This could indicate an interaction between the synthesis of peptidoglycan, the disruption of the cytoplasmic membrane and biofilm formation. Nevertheless, the IBF of all antibiotic combinations was superior to with single use of antibiotics. Additionally, the share of NBPs was higher.

Both aminoglycosides and quinolones are frequently used to treat infections caused by *P. aeruginosa.* The synergy of fosfomycin/aminoglycoside and fosfomycin/quinolone combinations against its biofilm has already been documented to some extent, using different biofilm models, antibiotic representatives of these groups and different isolate numbers, sometimes only one, on isolates that are predominantly sensitive to the tested agents [21,23,24,25,26,27,28]. Anderson et al. reported the eradication of *P. aeruginosa* biofilms using fosfomycin and tobramycin in combination [16]. The suggested synergy mechanism of quinolones and aminoglycosides with fosfomycin against planktonic cells of *P. aeruginosa* is that fosfomycin amends *P. aeruginosa* membrane permeability, leading to increased fluoroquinolone/aminoglycoside uptake. Although fosfomycin, ciprofloxacin and gentamicin were found to penetrate well into biofilms of *P. aeruginosa*, the lowest IBF rates were recorded for combinations with aminoglycosides and ciprofloxacin [21]. Nevertheless, combinations with both aminoglycosides and quinolones resulted in significantly higher levels of IBF. To further clarify the synergistic mechanism of these antibiotic combinations against biofilms, additional studies will be required.

We also observed a vast difference between the inhibitory properties of certain antibiotics, including fosfomycin, aminoglycosides, imipenem and ciprofloxacin, against different isolates, with higher OD_620_ values after exposure to antibiotics. One explanation for this effect could be the biofilm induction potential following exposure to subMIC concentrations of some antibiotics such as aminoglycosides, β-lactams and fluoroquinolones in *P. aeruginosa* isolates [2,22,32].

While a downgrading of the biofilm category was observed for some isolates, no statistically significant effect on biofilm disruption was observed with either the sole or combined use of antibiotics, suggesting the weak disruption potential of antibiotics for mature biofilms. One limitation of antibiotic usage against biofilms is the fact that when the treatments are initiated, the biofilm is already formed, suggesting the importance of biofilm prevention. Furthermore, antibiotics are more likely to be effective when used in the initial biofilm formation stages, while the bacterial cells are more metabolically active and thus more accessible for antibiotic therapy. Unfortunately, low antibiotic activity was exerted on already formed biofilms. However, a category downgrade indicates antibiofilm activity with potential that needs further investigation. Effects of combination therapy have largely been observed in the prevention of biofilm formation, rather than the disruption of biofilms. The use of a static or closed in vitro biofilm model provides insights into biofilm physiology but is insufficient in controlling environmental factors like nutrient flux and the capacity to wash out metabolic products, as well as host factors [16,23,33]. Dynamic or open systems provide conditions that better mimic those in vivo. Further studies using more clinical isolates and different biofilm models would provide additional insight into the impact of combination therapy in terms of biofilm prevention and disruption. Furthermore, a complete chequerboard analysis would provide a more comprehensive evaluation of antibiotic combination effects.

In conclusion, our results prove that the in vitro anti-biofilm effect of antibiotic combinations with fosfomycin, particularly in terms of the IBF, is considerably superior to the single use of antibiotics when using clinically achievable concentrations, even in situations where both antibiotics are not effective when used alone. Furthermore, according to these results, the IBF potential of combinations with β-lactam antibiotics and colistin is larger than that of combinations with protein synthesis inhibitors. The results obtained in this study could prove to be clinically valuable, particularly regarding biofilm prophylaxis of not only susceptible but also antibiotic-resistant isolates.

## 4. Materials and Methods

### 4.1. Bacterial Isolates

We collected forty-three clinical isolates of *P. aeruginosa* from various clinical specimens. Forty-one isolates were collected from six hospitals in Croatia: General Hospital Bjelovar, General Hospital Slavonski Brod, General Hospital Pula, University Hospital Centre Split, University Hospital Centre Osijek and University Hospital Centre Zagreb. Vitek 2 or MALDI-TOF MS was used to identify the isolates. Two of the isolates were kindly provided by G. M. Rossolini (Department of Microbiology and Virology, Careggi University Hospital, 50135 Florence, Italy). Antibiotic susceptibility was determined in previous studies carried out by Slade-Vitković et al. [34], and the isolates were categorized as multidrug-resistant (MDR) and/or extensively drug-resistant (XDR) according to Magiorakos et al. [35]. To define resistance, the fosfomycin MIC breakpoint value was set at >64 µg/mL [36]. Thirteen strong and eleven moderate biofilm producers were used to evaluate the anti-biofilm properties of fosfomycin alone and when combined with antipseudomonal antibiotics. All the isolates were MDR or XDR.

### 4.2. Quantitative Absorbance-Based Biofilm Measurement

#### 4.2.1. The Microtiter Plate Assay

For quantitative detection of the biofilms, the microtiter plate assay was employed [37]. Isolates were grown overnight on blood agar plates, and three to four colonies of each isolate were suspended in 5 mL of TSB (Tryptic Soy Broth). The cultures were incubated for 18 h at 36 °C. The tubes were shaken and diluted in 1:100 TSB. Then, 200 µL of the diluted culture was pipetted into 96-well plates and incubated at 36 °C for a further 24 h. After the incubation period, the medium was removed, and sterile distilled water was used to carefully wash the wells three times. The plates were air-dried at 56 °C for 60 min. To stain the biofilm, 250 µL of crystal violet solution was pipetted into each well. The dye was removed after 15 min, and the wells were cautiously washed using sterile distilled water. To dissolve the attached dye, 95% ethanol was added to each well, and the content was transferred into a flat-bottomed 96-well plate. Optical density was determined twice for each well using a microtiter plate reader at 620 nm. Sterile TSB was used as a negative control. Each experiment was performed in quadruplicate. The average OD_620_ values were determined for the tested isolates and for negative controls. To define the cut-off value (OD_c_), the following formula was used: OD_c_ = averageOD_negative control_ + (3 × SD_negative control_).

The isolates were categorized as follows: 4 × OD_c_ < OD = strong biofilm producer, 2 × OD_c_ < OD ≤ 4 × OD_c_ = moderate biofilm producer, OD_c_ < OD ≤ 2 × OD_c_ = weak biofilm producer, OD ≤ OD_c_ = non-biofilm producer [37].

#### 4.2.2. Inhibition of the Formation of and Eradication/Disruption of *P. aeruginosa* Biofilms

Inhibition of the formation of and eradication/disruption of the *P. aeruginosa* biofilms were evaluated using the crystal violet assay [37,38,39,40,41]. The cultures were exposed to eight antipseudomonal antibiotics alone and in combination. Peak plasma concentrations of non-protein bound drugs according to the literature were used: fosfomycin (395 µg/mL) [42], ceftazidime (170 µg/mL) [43], piperacillin/tazobactam (210/24 µg/mL), cefepime (131 µg/mL) [42], imipenem (55 µg/mL) [44], gentamicin (9 µg/mL) [45], amikacin (38 µg/mL) [42,46], ciprofloxacin (2.8 µg/mL) [42] and colistin (2.9 µg/mL) [47,48,49].

Biofilm formation inhibition was evaluated for 13 strong biofilm producers (P4, P5, P9, P11, P14, P19, P27, P30, P36, P39, P44, P46 and P57) and 11 moderate biofilm producers (P2, P8, P16, P29, P35 P37, P38, P41, P47, P48 and P56). Colonies were grown overnight on Columbia agar plates. Each isolate was suspended and diluted in TSB, as described above. Antibiotics, alone or in combination, were added to each diluted culture at the desired concentration. A total of 200 µL of each culture was transferred into the 96-well plates, and the plates were incubated for 24 h at 36 °C.

For the biofilm disruption assay, eight isolates with the highest OD_620_ values determined in the biofilm microtiter detection assay were used (P9, P14, P19, P30, P36, P44, P46 and P57). Biofilms were allowed to form for 24 h, as described above. After 24 h, the medium containing unattached bacteria was discarded, and sterile distilled water was used to gently wash the plate. The desired antibiotic concentrations, alone or in combination with fosfomycin, were prepared in sterile TSB and added to the microtiter plate wells, and the plates were incubated for 6 h and 24 h at 36 °C.

After the incubation period, the medium was removed from the plates used for both tests (inhibition and disruption assays), and the plates were carefully washed with sterile distilled water and allowed to dry. The biofilms were stained with crystal violet for 15 min, the unbound dye was removed, the wells were washed and the adhering dye was dissolved with ethanol. The biofilms were quantified in a spectrophotometer at 620 nm. Each measurement was performed in duplicate. TSB was used as a blank. Each experiment was performed in quadruplicate, with mean values and standard deviations calculated [38,39,40,41].

#### 4.2.3. Quantification of Biofilm Inhibition

Inhibition of biofilm formation (IBF) and biofilm disruption (BD) were calculated from the mean absorbance at 620 nm for each sample after antibiotic exposure according to the following formula: Biofilm_inhibition/disruption_ (%) = {(OD_untreated control_ − OD_treated sample_)/OD_untreated control_} × 100.

The results were expressed as percentages [29,50,51,52]. Means and standard deviations (SDs) for each sample were calculated and expressed with error bars, Figure 1 and Figure 2. To evaluate the biofilm inhibition potential of each antibiotic, the average biofilm inhibition rates of the antibiotics alone and in combination were determined and expressed as means and SDs.

To further evaluate the inhibition potential of the antibiotics and antibiotic combinations, the category changes in 13 strong and 11 moderate biofilm-producing isolates were assessed.

### 4.3. Statistical Analysis

All the experiments were performed in quadruplicate for each strain. Data were expressed as means with standard deviations. To determine significant differences between groups, the Friedman and Wilcoxon Signed Rank non-parametric tests were used. SPSS software (https://www.ibm.com/spss, accessed on 7 June 2024) was used for the statistical analysis. The data met the test assumptions of the tests. A *p*-value of less than 0.05 was considered statistically significant.

## Figures and Tables

**Table 1:** MIC values and resistance phenotypes of *P. aeruginosa* isolates.

Isolate		MIC (µg/mL) ^a^									Category ^b^
	FOM	CAZ	FEP	TZP	IMI	MEM	GM	AM	CIP	COL	
P1	128	64	32	128	128	>128	>128	8	32	1	XDR
P2	>256	>128	>128	>128	64	32	>128	128	64	2	XDR
P3	64	>128	>128	>128	>128	>128	>128	32	64	2	XDR
P4	128	128	32	32	128	>128	>128	128	32	1	XDR
P5	128	128	16	64	128	>128	1	2	2	1	XDR
P6	>256	64	32	64	128	>128	32	128	64	1	XDR
P7	256	64	32	128	128	>128	32	128	32	1	XDR
P8	64	32	16	64	128	>128	32	32	32	1	XDR
P9	128	64	32	32	32	>128	32	128	32	2	XDR
P11	8	>128	32	>128	>128	>128	>128	2	>128	1	MDR
P12	32	>128	32	32	128	>128	16	64	64	1	XDR
P14	>128	>128	>128	>128	>128	>128	>128	64	>128	2	XDR
P15	128	>128	64	>128	>128	>128	>128	128	>128	2	XDR
P16	16	64	32	64	>128	>128	8	32	>128	2	XDR
P17	128	32	16	16	32	16	>128	16	2	2	MDR
P18	>128	16	16	32	>128	16	8	4	2	4	XDR
P19	32	>128	32	>128	>128	16	>128	4	16	2	MDR
P27	128	16	32	16	8	1	256	8	0.5	2	MDR
P28	128	64	4	8	4	4	4	8	0.25	2	MDR
P29	128	8	16	16	2	1	>128	128	>128	2	MDR
P30	64	32	32	16	16	16	>128	4	>128	2	MDR
P33	128	16	16	16	32	16	8	4	2	2	MDR
P35	64	128	64	32	>128	64	>128	128	128	2	XDR
P36	32	>128	32	16	4	64	2	16	32	2	MDR
P37	64	32	8	16	16	16	32	32	32	4	MDR
P38	64	>128	32	128	16	16	>128	32	32	2	XDR
P39	128	>128	32	16	16	16	32	64	64	2	MDR
P40	128	64	32	16	16	16	>128	64	64	2	MDR
P41	>128	64	16	16	8	16	64	32	64	2	MDR
P43	4	>128	>128	>128	16	16	>128	64	64	4	XDR
P44	4	>128	64	>128	64	16	>128	32	64	4	XDR
P45	64	64	16	32	16	16	8	8	0.5	2	MDR
P46	2	128	64	256	16	16	>128	64	64	4	XDR
P47	4	>128	>128	>128	128	32	>128	64	128	1	XDR
P48	2	>128	64	>128	64	8	>128	64	128	2	XDR
P49	64	16	16	32	32	8	4	8	0.5	1	MDR
P50	4	>128	>128	>128	64	16	>128	64	64	2	XDR
P51	4	>128	>128	256	64	32	>128	128	64	4	XDR
P52	16	>128	64	>128	32	16	>128	32	64	2	XDR
P53	>128	64	32	16	32	16	>128	16	2	2	MDR
P54	>128	16	8	8	16	16	4	16	0.125	2	MDR
P56	>128	>256	>256	64	0.5	1	>256	64	64	2	MDR
P57	>128	>256	128	64	128	64	>256	128	64	2	XDR

^a^ MIC (minimal inhibitory concentration) was determined using microdilution method, with the exception of fosfomycin, for which agar dilution method was used. MIC of FOM ≤ 64 µg/mL was considered susceptible, MIC of FOM > 64 µg/mL was considered resistant. ^b^ MDR (multidrug-resistant): non-susceptible to ≥1 agent in ≥3 antimicrobial categories; XDR (extensively drug-resistant): non-susceptible to ≥1 agent in all but ≤2 categories. Abbreviations: fosfomycin (FOM), ceftazidime (CAZ), piperacillin/tazobactam (TZP), cefepime (FEP), meropenem (MEM), imipenem (IMI), gentamicin (GM), amikacin (AM), ciprofloxacin (CIP) and colistin (COL).

**Table 2:** Average IBF rates of antibiotics alone and in combination, expressed as means and SD.

Atb	Average IBF				
	Single Use %	SD	Combination %	SD	*p*
FOM	14.9	±67.75			
CAZ	56.5	±29.96	81.0	±8.39	<0.001
FEP	77.1	±18.35	81.8	±7.35	<0.001
TZP	36.8	±41.78	78.8	±13.19	<0.001
IMI	15.2	±58.14	74.1	±21.16	<0.001
GM	−20.8	±59.99	39.9	±68.82	<0.001
CIP	−24.2	±61.78	52.8	±47.19	<0.001
COL	75.6	±20.73	82.3	±6.41	<0.001
AM	−18.3	±91.26	68.1	±39.66	<0.001

Abbreviations: inhibition of biofilm formation (IBF), atb (antibiotic), fosfomycin (FOM), ceftazidime (CAZ), piperacillin/tazobactam (TZP), cefepime (FEP), imipenem (IMI), gentamicin (GM), amikacin (AM), ciprofloxacin (CIP), colistin (COL), standard deviation (SD), *p*-value (*p*).

**Table 3:** Average calculated OD_620_ values of 24 h old biofilms after 6 h of exposure to single and combined antibiotics and biofilm disruption rates, expressed as means with standard deviation.

	OD_atb_	OD_comb_	BD %	SD	BD_comb_ %	SD
CAZ	0.607594	0.521313	4.75	±14.80	18.81	±9.72
FEP	0.491531	0.495109	21.00	±12.42	22.35	±12.15
TZP	0.648563	0.552984	−4.64	±12.71	13.24	±13.23
IMI	0.490469	0.467781	23.20	±9.57	27.59	±12.48
GM	0.55825	0.539375	11.93	±9.69	14.85	±15.81
CIP	0.644125	0.572797	−4.15	±10.95	10.26	±14.79
COL	0.465239	0.495609	26.72	±17.43	23.94	±12.14
AM	0.580547	0.526031	10.77	±10.17	19.48	±13.24
FOM	0.562453		11.51	±11.28		

Abbreviations: optical density (OD), biofilm disruption (BD), standard deviation (SD), atb (antibiotic), fosfomycin (FOM), ceftazidime (CAZ), piperacillin/tazobactam (TZP), cefepime (FEP), imipenem (IMI), gentamicin (GM), amikacin (AM), ciprofloxacin (CIP), colistin (COL).

**Table 4:** Average calculated OD_620_ values of 24 h old biofilms after 24 h of exposure to single and combined antibiotics and biofilm disruption rates, expressed as means with standard deviation.

	OD_atb_	OD_comb_	BD %	SD	BD_comb_ %	SD
CAZ	0.625203	0.670141	8.49	±16.87	2.71	±19.91
FEP	0.593656	0.651391	11.91	±12.37	2.77	±12.91
TZP	0.561375	0.648823	16.70	±11.89	3.95	±10.97
IMI	0.590844	0.568	10.82	±11.63	14.02	±13.31
GM	0.586094	0.640443	13.68	±6.44	4.01	±9.45
CIP	0.601026	0.597516	10.70	±8.51	9.94	±17.19
COL	0.625568	0.62611	8.24	±8.52	7.54	±12.06
AM	0.636359	0.572573	6.61	±13.19	15.38	±11.91
FOM	0.678063		−3.81	±23.85		

Abbreviations: optical density (OD), biofilm disruption (BD), standard deviation (SD), atb (antibiotic), fosfomycin (FOM), ceftazidime (CAZ), piperacillin/tazobactam (TZP), cefepime (FEP), imipenem (IMI), gentamicin (GM), amikacin (AM), ciprofloxacin (CIP), colistin (COL).

## Data Availability

The data presented in the study are available on request from the corresponding author.

## References

[1] Zakhour J., Sharara S.L., Hindy J.R., Haddad S.F., Kanj S.S. (2022). Antimicrobial Treatment of *Pseudomonas aeruginosa* Severe Sepsis. Antibiotics.

[2] Olivares E., Badel-Berchoux S., Provot C., Prévost G., Bernardi T., Jehl F. (2020). Clinical Impact of Antibiotics for the Treatment of *Pseudomonas aeruginosa* Biofilm Infections. Front. Microbiol..

[3] Yousef Memar M., Adibkia K., Farajnia S., Kafil H.S., Khalili Y., Azargun R., Ghotaslou R. (2021). In-vitro Effect of Imipenem, Fosfomycin, Colistin, and Gentamicin Combination against Carbapenem-resistant and Biofilm-forming *Pseudomonas aeruginosa* Isolated from Burn Patients. Iran. J. Pharm. Res..

[4] Černohorská L., Votava M. (2008). Antibiotic synergy against biofilm-forming *Pseudomonas aeruginosa*. Folia Microbiol..

[5] Ghorbani H., Memar M.Y., Sefidan F.Y., Yekani M., Ghotaslou R. (2017). In vitro synergy of antibiotic combinations against planktonic and biofilm *Pseudomonas aeruginosa*. GMS Hyg. Infect. Control.

[6] Costa G.A., Rossatto F.C.P., Medeiros A.W., Paula A., Correa F., Brandelli A., Frazzon A.P.G., Motta A.D.S.D. (2018). Evaluation antibacterial and antibiofilm activity of the antimicrobial peptide P34 against *Staphylococcus aureus* and *Enterococcus faecalis*. An. Acad. Bras. Cienc..

[7] Rojo-Molinero E., Macià M.D., Rubio R., Moyà B., Cabot G., López-Causapé C., Oliver A. (2016). Sequential Treatment of Biofilms with Aztreonam and Tobramycin Is a Novel Strategy for Combating *Pseudomonas aeruginosa* Chronic Respiratory Infections. Antimicrob. Agents Chemother..

[8] Ciofu O., Rojo-Molinero E., Macià M.D., Oliver A. (2017). Antibiotic treatment of biofilm infections. Apmis.

[9] Penesyan A., Paulsen I.T., Gillings M.R., Kjelleberg S., Manefield M.J. (2020). Secondary Effects of Antibiotics on Microbial Biofilms. Front. Microbiol..

[10] Raz R., Raz P.R. (2012). Fosfomycin: An old—New antibiotic. Clin. Microbiol. Infect..

[11] Silver L.L. (2017). Fosfomycin: Mechanism and Resistance. Cold Spring Harb. Perspect. Med..

[12] Walsh C.C., Landersdorfer C.B., McIntosh M.P., Peleg A.Y., Hirsch E.B., Kirkpatrick C.M., Bergen P.J. (2016). Clinically relevant concentrations of fosfomycin combined with polymyxin, B.; tobramycin or ciprofloxacin enhance bacterial killing of *Pseudomonas aeruginosa*, but do not suppress the emergence of fosfomycin resistance. J. Antimicrob. Chemother..

[13] Díez-Aguilar M., Cantón R. (2019). New microbiological aspects of fosfomycin. Rev. Española Quimioter..

[14] Roussos N., Karageorgopoulos D.E., Samonis G., Falagas M.E. (2009). Clinical significance of the pharmacokinetic and pharmacodynamic characteristics of fosfomycin for the treatment of patients with systemic infections Clinical signifi-cance of the pharmacokinetic and pharmacodynamic characteristics of fosfomycin for the treatment of patients with systemic infections. Int. J. Antimicrob. Agents.

[15] Michalopoulos A.S., Falagas M.E. (2011). Colistin: Recent data on pharmacodynamics properties and clinical efficacy in critically ill patients. Ann. Intensive Care.

[16] Anderson G.G., Kenney T.F., MacLeod D.L., Henig N.R., O’Toole G.A. (2013). Eradication of *Pseudomonas aeruginosa* biofilms on cultured airway cells by a fosfomycin/tobramycin antibiotic combination. Pathog. Dis..

[17] Chai D., Liu X., Wang R., Bai Y., Cai Y. (2016). Efficacy of Linezolid and Fosfomycin in Catheter-Related Biofilm Infection Caused by Methicillin-Resistant *Staphylococcus aureus*. Biomed. Res. Int..

[18] González M.J., Da Cunda P., Notejane M., Zunino P., Scavone P., Robino L. (2019). Fosfomycin tromethamine activity on biofilm and intracellular bacterial communities produced by uropathogenic *Escherichia coli* isolated from patients with urinary tract infection. Pathog. Dis..

[19] Walsh C.C., McIntosh M.P., Peleg A.Y., Kirkpatrick C.M., Bergen P.J. (2015). In vitro pharmacodynamics of fosfomycin against clinical isolates of *Pseudomonas aeruginosa*. J. Antimicrob. Chemother..

[20] Samonis G., Maraki S., Karageorgopoulos D.E., Vouloumanou E.K., Falagas M.E. (2012). Synergy of fosfomycin with carbapenems, colistin, netilmicin, and tigecycline against multidrug-resistant *Klebsiella pneumoniae, Escherichia coli*, and *Pseudomonas aeruginosa* clinical isolates. Eur. J. Clin. Microbiol. Infect. Dis..

[21] Wang L., Di Luca M., Tkhilaishvili T., Trampuz A., Gonzalez Moreno M. (2019). Synergistic Activity of Fosfomycin, Ciprofloxacin, and Gentamicin Against *Escherichia coli* and *Pseudomonas aeruginosa* Biofilms. Front. Microbiol..

[22] Tré-Hardy M., Nagant C., El Manssouri N., Vanderbist F., Traore H., Vaneechoutte M., Dehaye J.P. (2010). Efficacy of the Combination of Tobramycin and a Macrolide in an In Vitro *Pseudomonas aeruginosa* Mature Biofilm Model. Antimicrob. Agents Chemother..

[23] Díez-Aguilar M., Morosini M.I., Köksal E., Oliver A., Ekkelenkamp M., Cantón R. (2018). Use of Calgary and Microfluidic BioFlux Systems To Test the Activity of Fosfomycin and Tobramycin Alone and in Combination against Cystic Fibrosis *Pseudomonas aeruginosa* Biofilms. Antimicrob. Agents Chemother..

[24] Cai Y., Fan Y., Wang R., An M.M., Liang B.B. (2009). Synergistic effects of aminoglycosides and fosfomycin on *Pseudomonas aeruginosa* in vitro and biofilm infections in a rat model. J. Antimicrob. Chemother..

[25] Mikuniya T., Kato Y., Kariyama R., Monden K., Hikida M., Kumon H. (2005). Synergistic effect of fosfomycin and fluoroquinolones against *Pseudomonas aeruginosa* growing in a biofilm. Acta Med. Okayama.

[26] Mikuniya T., Kato Y., Ida T., Maebashi K., Monden K., Kariyama R., Kumon H. (2007). Treatment of *Pseudomonas aeruginosa* biofilms with a combination of fluoroquinolones and fosfomycin in a rat urinary tract infection model. J. Infect. Chemother..

[27] Monden K., Ando E., Iida M., Kumon H. (2002). Role of fosfomycin in a synergistic combination with ofloxacin against *Pseudomonas aeruginosa* growing in a biofilm. J. Infect. Chemother..

[28] Kumon H., Ono N., Iida M., Nickel J.C. (1995). Combination Effect of Fosfomycin and Ofloxacin against *Pseudomonas aeruginosa* Growing in a Biofilm. Antimicrob. Agents Chemother..

[29] Su T., He J., Li N., Liu S., Xu S., Gu L. (2020). A Rational Designed PslG with Normal Biofilm Hydrolysis and Enhanced Resistance to Trypsin-Like Protease Digestion. Front. Microbiol..

[30] Boncompagni S.R., Micieli M., Di Maggio T., Aiezza N., Antonelli A., Giani T., Rossolini G.M. (2022). Activity of fosfomycin/colistin combinations against planktonic and biofilm Gram-negative pathogens. J. Antimicrob. Chemother..

[31] Agyeman A.A., López-Causapé C., Rogers K.E., Lucas D.D., Cortés-Lara S., Gomis-Font M.A., Landersdorfer C.B. (2023). Ceftolozane/tazobactam plus tobramycin against free-floating and biofilm bacteria of hypermutable *Pseudomonas aeruginosa* epidemic strains: Resistance mechanisms and synergistic activity. Int. J. Antimicrob. Agents.

[32] Habash M.B., Park A.J., Vis E.C., Harris R.J., Khursigara C.M. (2014). Synergy of silver nanoparticles and aztreonam against *Pseudomonas aeruginosa* PAO1 biofilms. Antimicrob. Agents Chemother..

[33] Lebeaux D., Chauhan A., Rendueles O., Beloin C. (2013). From in vitro to in vivo Models of Bacterial Biofilm-Related Infections. Pathogens.

[34] Slade-Vitković M., Bedenić B., Bielen L., Batarilo I., Kibel S., Maravić-Vlahoviček G. (2023). In vitro killing of multidrug/extensively drug-resistant Pseudomonas aeruginosa by fosfomycin alone or in combination with antipseudomonal antibiotics. J. Chemother..

[35] Magiorakos A.P., Srinivasan A., Carey R.B., Carmeli Y., Falagas M.E., Giske C.G., Monnet D.L. (2012). Multidrug-resistant, extensively drug-resistant and pandrug-resistant bacteria: An international expert proposal for interim standard definitions for acquired resistance. Clin. Microbiol. Infect..

[36] López-Montesinos I., Horcajada J.P. (2019). Oral and intravenous fosfomycin in complicated urinary tract infections. Rev. Esp. Quimioter..

[37] Stepanović S., Vuković D., Hola V., Di Bonaventura G., Djukić S., Ćirković I., Ruzicka F. (2007). Quantification of biofilm in microtiter plates: Overview of testing conditions and practical recommendations for assessment of biofilm production by staphylococci. Apmis.

[38] Liu Y., Wu L., Han J., Dong P., Luo X., Zhang Y., Zhu L. (2020). Inhibition of Biofilm Formation and Related Gene Expression of *Listeria monocytogenes* in Response to Four Natural Antimicrobial Compounds and Sodium Hypochlorite. Front. Microbiol..

[39] Warraich A.A., Mohammed A.R., Perrie Y., Hussain M., Gibson H., Rahman A. (2020). Evaluation of anti-biofilm activity of acidic amino acids and synergy with ciprofloxacin on Staphylococcus aureus biofilms. Sci. Rep..

[40] Bernal-Mercado A., Vazquez-Armenta F., Tapia-Rodriguez M., Islas-Osuna M., Mata-Haro V., Gonzalez-Aguilar G., Ayala-Zavala J.F. (2018). Comparison of single and combined use of catechin, protocatechuic, and vanillic acids as antioxidant and antibacterial agents against uropathogenic *Escherichia coli* at planktonic and biofilm levels. Molecules.

[41] Wickremasinghe H., Yu H.H., Azad M.A.K., Zhao J., Bergen P.J., Velkov T., Li J. (2021). Clinically relevant concentrations of polymyxin B and meropenem synergistically kill multidrug-resistant *Pseudomonas aeruginosa* and minimize biofilm formation. Antibiotics.

[42] Monogue M.L., Nicolau D.P. (2018). Antibacterial activity of ceftolozane/tazobactam alone and in combination with other antimicrobial agents against MDR *Pseudomonas aeruginosa*. J. Antimicrob. Chemother..

[43] FDA (2021). Ceptaz (Ceftazidime for Injection) [Internet].

[44] FDA (2016). Primaxin (Imipenem and Cilastatin) for Injection, for Intravenous Use.

[45] FDA (2013). Fresenius Kabi USA, LCC. Gentamicin Injection. https://www.accessdata.fda.gov/drugsatfda_docs/label/2014/062366s033lbl.pdf.

[46] FDA (2021). Amikacin Sulfate [Internet].

[47] Car H. (2020). In Vitro Synergy and Postantibiotic Effect of Colistin Combinations with Meropenem and Vancomycin against Gram Negative Bacteria with Multiple Carbapenem Resistance Mechanisms. Ph.D. Thesis.

[48] Markou N., Markantonis S.L., Dimitrakis E., Panidis D., Boutzouka E., Karatzas S., Baltopoulos G. (2008). Colistin serum concentrations after intravenous administration in critically ill patients with serious multidrug-resistant, gram-negative bacilli infections: A prospective, open-label, uncontrolled study. Clin. Ther..

[49] Moni M., Sudhir S., Dipu T.S., Mohamed Z., Prabhu B.P., Edathadathil F., Menon V.P. (2020). Clinical efficacy and pharmacokinetics of colistimethate sodium and colistin in critically ill patients in an Indian hospital with high endemic rates of multidrug-resistant Gram-negative bacterial infections: A prospective observational study. Int. J. Infect. Dis..

[50] Das M.C., Sandhu P., Gupta P., Rudrapaul P., De U.C., Tribedi P., Bhattacharjee S. (2016). Attenuation of *Pseudomonas aeruginosa* biofilm formation by Vitexin: A combinatorial study with azithromycin and gentamicin. Sci. Rep..

[51] Abu El-Wafa W.M., Ahmed R.H., Ramadan M.A.H. (2020). Synergistic effects of pomegranate and rosemary extracts in combination with antibiotics against antibiotic resistance and biofilm formation of *Pseudomonas aeruginosa*. Braz. J. Microbiol..

[52] Shinde S., Lee L.H., Chu T. (2021). Inhibition of Biofilm Formation by the Synergistic Action of EGCG-S and Antibiotics. Antibiotics.

